# SIRT1 but not its increased expression is essential for lifespan extension in caloric-restricted mice

**DOI:** 10.1111/acel.12151

**Published:** 2013-11-19

**Authors:** Evi M Mercken, Jia Hu, Susan Krzysik-Walker, Min Wei, Ying Li, Michael W McBurney, Rafael de Cabo, Valter D Longo

**Affiliations:** 1 Translational Gerontology Branch, National Institute on Aging, National Institutes of HealthBaltimore, MD, 21224, USA; 2 Davis School of Gerontology and Department of Biological Sciences, University of Southern CaliforniaLos Angeles, CA, 90089-2520, USA; 3 Laboratory of Clinical Investigation, National Institute on Aging, National Institutes of HealthBaltimore, MD, 21224, USA; 4 Department of Medicine, Ottawa Hospital Research Institute, University of OttawaOttawa, Ontario, K1H 8L6, Canada

**Keywords:** anti-aging, caloric restriction, lifespan, SIRT1

## Abstract

The SIRT1 deacetylase is one of the best-studied putative mediators of some of the anti-aging effects of calorie restriction (CR), but its role in CR-dependent lifespan extension has not been demonstrated. We previously found that mice lacking both copies of SIRT1 displayed a shorter median lifespan than wild-type mice on an *ad libitum* diet. Here, we report that median lifespan extension in CR heterozygote SIRT1^+/−^ mice was identical (51%) to that observed in wild-type mice, but SIRT1^+/−^ mice displayed a higher frequency of certain pathologies. Although larger studies in additional genetic backgrounds are needed, these results provide strong initial evidence for the requirement of SIRT1 for the lifespan extension effects of CR, but suggest that its high expression is not required for CR-induced lifespan extension.

## Introduction

The discovery that sirtuins influence yeast replicative lifespan more than 10 years ago sparked wide interest about their biological role in aging, age-related diseases, and calorie restriction (CR)-mediated lifespan extension. Although the contribution of SIRT1 in both longevity and CR-mediated lifespan extension in lower organisms is a subject of debate (Kaeberlein *et al*., 2004; Fabrizio *et al*., 2005), there is evidence that SIRT1 plays an important role in delaying or ameliorating the onset of some age-associated diseases (Herranz *et al*., 2010). However, the role of SIRT1 in the mediation of CR-dependent lifespan extension in mammals remains elusive and controversial (Fontana *et al*., 2010; Hayflick, 2010).

**Figure 1:**
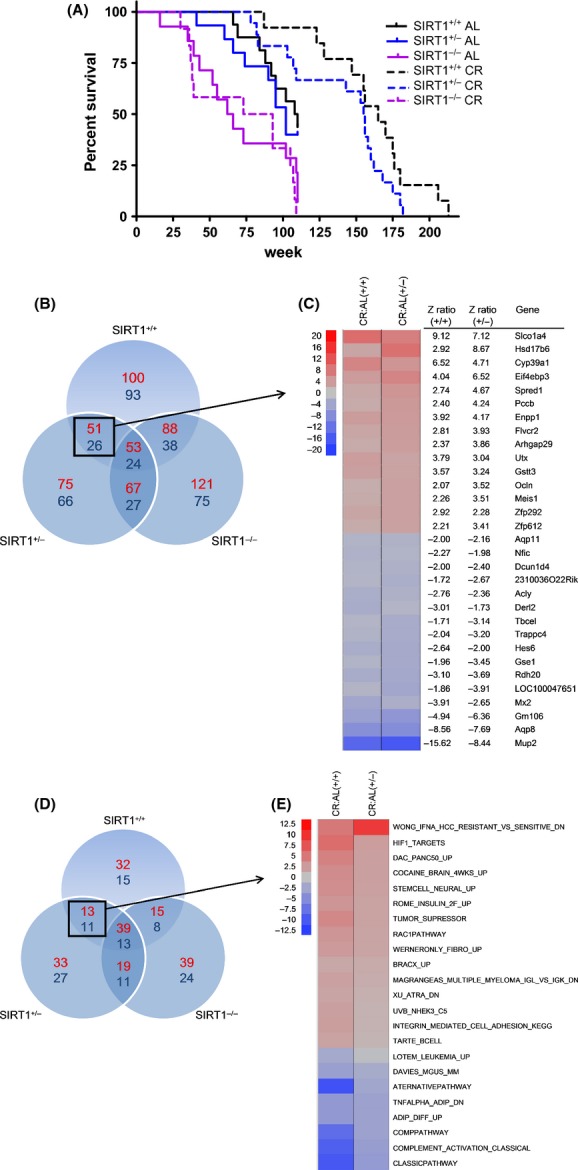
Genetic manipulation of SIRT1 increases lifespan. (A) Kaplan–Meier survival curves of the indicated genotypes under *ad libitum* (AL) and 40% CR-fed conditions, with *P*-values calculated using the log rank test. A significant increase in median and maximal lifespan was observed in SIRT1^+/+^ and SIRT1^+/−^ versus SIRT1^−/−^ mice (*P* < 0.001 and *P* < 0.01, respectively (median), and *P* < 0.001 for both comparisons (maximal)). No statistically significant difference in median lifespan was observed between SIRT1^+/+^ and SIRT1^+/−^ (*P* = 0.108), whereas maximal lifespan tended to be extended in SIRT1^+/+^ compared with SIRT1^+/−^ mice (*P* = 0.067). No statistical differences in median lifespan between AL- and CR-fed SIRT1^−/−^ mice were detected. Venn diagram showing the gene (B) and pathway (D) interactions between the different diets and genotypes (upregulated and downregulated genes/pathways are shown in red and blue, respectively). The most highly significant upregulated and downregulated common genes (C) and pathways (E) between SIRT1^+/+^ and SIRT1^+/−^ are shown.

To determine whether SIRT1 modulates the effects of CR on aging in mammals, we subjected 2–5-month-old mice of three different genotypes (SIRT1^+/+^, SIRT1^+/−^, and SIRT1^−/−^) that had normal, reduced, or no expression of SIRT1 to either 40% CR or *ad libitum* (AL) diets. SIRT1^−/−^ mice on CR displayed a short lifespan (Fig. 1A) (Boily *et al*., 2008; Li *et al*., 2008), whereas CR extended the lifespan of both SIRT1^+/−^ and SIRT1^+/+^ mice by 51% (Fig. 1A and Table S1–S2). The deletion of one SIRT1 allele resulted in a trend for decreased maximum lifespan under CR conditions (Fig. 1A). These results indicate that high expression of SIRT1 is not required for the lifespan extension caused by CR.

**Figure 2:**
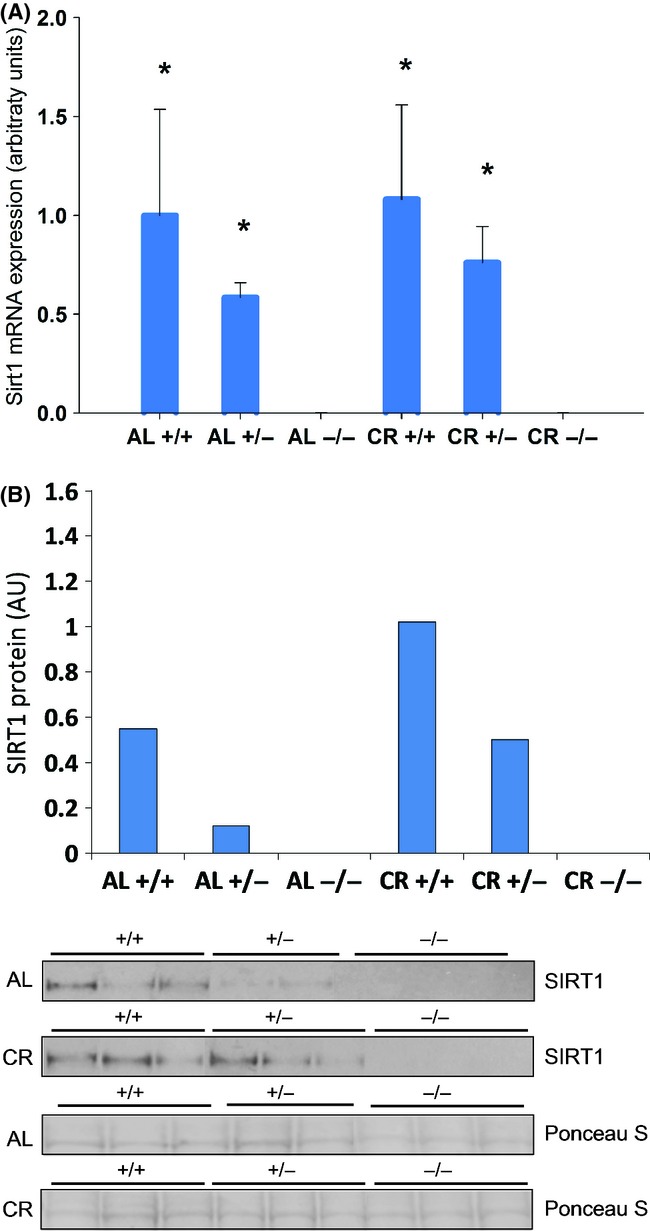
SIRT1 expression is increased in response to CR. (A) SIRT1 mRNA levels were measured by RT-qPCR. Liver homogenates from *ad libitum* (AL) and 40% CR-fed SIRT1^+/+^, SIRT1^+/−^, and SIRT1^−/−^ mice were tested. A significant increase in SIRT1 expression in CR and AL-fed SIRT1^+/+^ and SIRT1^+/−^ mice was observed as compared to SIRT1^−/−^ mice (*P < 0.05). *n* = 3 for all groups. Error bars indicate SEM. (B) SIRT1 protein levels were determined by Western blotting. *n* = 2–3 for all groups.

To address how SIRT1 heterozygosity affects its expression, we measured SIRT1 expression in the liver. Our data show that, under both dietary regimes, there was a genotype-associated proportional decrease in SIRT1 expression both at the mRNA and protein levels (Fig. 2). SIRT1 expression showed a trend of increase in CR compared with AL animals (Fig. 2), in agreement with the increase in SIRT1 protein levels reported for mice, rats, and humans (Baur *et al*., 2012).

To investigate whether reduced SIRT1 expression in the SIRT1^+/−^ mice affected the levels of SIRT1-dependent genes altered by CR in the liver, we performed microarray analyses. A comparison of the gene expression profiles from SIRT1^+/+^ and SIRT1^+/−^ genotypes led to the identification of 77 SIRT1-dependent genes (51 upregulated, 26 downregulated, a total change of 19%) and 24 pathways (13 upregulated, 11 downregulated, a total change of 18%) that were altered in response to CR (Fig. 1B–E).

Decreased SIRT1 expression resulted in a dose-dependent change in many genes and pathways (Fig. S1, for a complete list of genes see Table S3). Many of the CR- and SIRT1-affected genes are involved in metabolic homeostasis, cell cycle, inflammation, and stress response/detoxification pathways, areas in which SIRT1 is known to have beneficial effects. Among them, GCK, IRS2, and several members of the major urinary protein (MUPs) family were altered by CR in a SIRT1 gene-dose-dependent manner. These findings were further confirmed using RT-qPCR (Fig. S2A). GCK, a key enzyme in the glycogen biosynthesis pathway and a proposed target for the development of CR mimetics (Ingram & Roth, 2011), was downregulated in response to CR. Concurrent with these changes, there was an upregulation of IRS2, an integrator of insulin receptor signals that coordinates metabolism (Dong *et al*., 2006). Furthermore, MUP1, which was recently been shown to play an important role in glucose homeostasis (Zhou *et al*., 2009), was downregulated in response to CR on both the mRNA (Fig. S2A) and protein (Fig. S2B) levels. The presence of only one SIRT1 allele was sufficient to cause a major reduction in the expression of many but not all of the CR-affected genes that depend on SIRT1. One could speculate that the genes not affected by the SIRT1^+/−^ genotype may be responsible for the identical median lifespan observed in the CR SIRT1^+/−^ and wild-type mice. However, the more likely explanation is that an increased activity or expression of SIRT1 during CR is not required for the anti-aging effects of CR. With our current data set, it remains unclear which of the many SIRT1-affected genes/pathways contribute to the beneficial effects of CR or which particular tissue(s) is/are mainly responsible for the metabolic/health effects induced by CR. It is also important to note that several SIRT1-independent genes were also altered by CR (Table S4 and S5).

Although CR SIRT1^+/−^ mice were long-lived, they displayed more frequent defects/pathologies (44% incidence), including eye infections or redness, and abnormal growth compared with CR SIRT1^+/+^ mice (31% incidence) (Table S6). Previous studies have shown that SIRT1-knockout mice displayed defective metabolism, and CR-mediated physiological responses (i.e., increased physical activity) were abrogated (Chen *et al*., 2005; Boily *et al*., 2008). These data confirm the requirement to some degree of SIRT1 expression for the beneficial effects of CR. One point of contention is that SIRT1^−/−^ mice may die early because they are sick independently of the prolongevity effects of CR. However, CR-mediated lifespan extension is generally observed after 20 months of age in normal mice (Bartke *et al*., 2001). Here, the longest-living SIRT1^−/−^ animals (20% of the initial cohort) were approximately 25 months of age and still were refractory to lifespan extension following CR. These results suggest that some SIRT1 activity or protein levels are required for the effects of CR on health span, possibly because it regulates some of the genes and pathways that are essential for the CR-dependent metabolic responses.

Moderate overexpression of SIRT1 in mice on a standard diet has no effect on overall lifespan, yet health span is improved (Herranz *et al*., 2010). It is likely that, similar to CR, SIRT1 elicits some beneficial effects on overall health (Bordone *et al*., 2007). One possible reason for the absence of lifespan extension in SIRT1-overexpressing mice is that these animals are particularly prone to nonmetabolically related diseases (i.e., lymphoma), independent of SIRT1 dosage (Herranz *et al*., 2010).

## Supporting Information

**Fig. S1** Parametric analysis of gene-set enrichment (PAGE) was performed on microarray data from SIRT1^+/+^, SIRT1^+/−^ and SIRT1^−/−^ mice fed *ad libitum* (AL) or subjected to 40% CR. **Fig. S2** Genes affected by CR in a SIRT1 gene-dose dependent manner. (A) IRS2 GCK, and MUP1 mRNA levels were measured by RT-qPCR in liver homogenates from ad libitum (AL) and 40% CR-fed SIRT1^+/+^, SIRT1^+/−^ and SIRT1^−/−^ mice. n = 3 for all groups (*p < 0.05 compared to SIRT1^+/+^). **Table S1** Numbers and survival characteristics of SIRT1^+/+^, SIRT1^+/−^ and SIRT1^−/−^ ad libitum (AL) and calorie-restricted (CR) mice. **Table S2** Significance for all pairwise comparisons (diet, genotype and sex) using log rank test. **Table S3** Z-scores of top 100 genes altered by CR and sorted against SIRT1^−/−^ mice. **Table S4** List of genes affected by CR in a SIRT1-independent manner. **Table S5** RT-qPCR validation of genes affected by CR in a SIRT1-independent manner. **Table S6** Pathologies and abnormalities in old mice (> 24 months). **Table S7** RT-qPCR validation of microarray data. **Table S8** Primer sequences used for quantitative PCR analysis.
