# Supplementary material for: SIRT1 but not its increased expression is essential for lifespan extension in caloric-restricted mice
Source: Aging Cell. 2013 Nov 19;13(1):193–6. doi: 10.1111/acel.12151 (PMC3907112; doi:10.1111/acel.12151)
Supplement: Supplementary file 1 — Fig. S1 Parametric analysis of gene-set enrichment (PAGE) was performed on microarray data from SIRT1+/+, SIRT1+/− and SIRT1−/− mice fed ad libitum (AL) or subjected to 40% CR. Fig. S2 Genes affected by CR in a SIRT1 gene-dose dependent manner. (A) IRS2 GCK, and MUP1 mRNA levels were measured by RT-qPCR in liver homogenates from ad libitum (AL) and 40% CR-fed SIRT1+/+, SIRT1+/− and SIRT1−/− mice. n = 3 for all groups (*p < 0.05 compared to SIRT1+/+). Table S1 Numbers and survival characteristics of SIRT1+/+, SIRT1+/− and SIRT1−/− ad libitum (AL) and calorie-restricted (CR) mice. Table S2 Significance for all pairwise comparisons (diet, genotype and sex) using log rank test. Table S3 Z-scores of top 100 genes altered by CR and sorted against SIRT1−/− mice. Table S4 List of genes affected by CR in a SIRT1-independent manner. Table S5 RT-qPCR validation of genes affected by CR in a SIRT1-independent manner. Table S6 Pathologies and abnormalities in old mice (> 24 months). Table S7 RT-qPCR validation of microarray data. Table S8 Primer sequences used for quantitative PCR analysis. [file acel0013-0193-sd1.docx]

# Online Supporting Information for:

**SIRT1 but not its increased expression is essential for lifespan extension in caloric restricted mice.**

Evi M. Mercken,^1†^ Jia Hu,^2,3†^ Susan Krzysik-Walker,^4^ Min Wei^2^, Ying Li,^2^ Michael W. McBurney,^5^ Rafael de Cabo,^1*^ Valter D. Longo^2,3*^

^†^These authors contributed equally to this research.

^*^Corresponding authors: [vlongo@usc.edu](mailto:vlongo@usc.edu) (V.D.L.); [decabora@grc.nia.nih.gov](mailto:decabora@grc.nia.nih.gov) (R.de C.).

This pdf file includes:

**Experimental Procedures**

**Fig. S1-S2**

**Tables S1 to S8**

**References (15-17)**

**Experimental Procedures**

***Animals*** SIRT1^+/+^, SIRT1^+/-^ and SIRT1^-/-^ genotypes have been described previously ([McBurney *et al.* 2003](#_ENREF_3)). Three to five-month-old mice were single-caged and fed ad libitum (AL) or restricted to 60% of AL group (CR) with NIH-31 standard chow or the NIH31/NIA-fortified food (7109, Harlan Teklad), respectively. Water was available ad libitum for all mice. When mice were observed to have lesions, developed clinical symptoms or continuous body weight loss, the veterinarian made the determination of whether euthanasia should be performed. The animal protocol has been approved by USC IACUC (Institutional Animal Care and Use Committee).

***Microarray analysis*** Mice from each genotypic group (SIRT1^+/+^, SIRT1^+/-^ and SIRT1^-/-^; male/female, 13 to 16 months old, n=2-4 per group) on ad libitum (AL) or 40% caloric restriction (CR) were sacrificed, and the livers were collected and flash frozen. RNA was extracted using Trizol reagent (Invitrogen, Carlsbad, CA) following the manufacturer’s instructions and hybridized to MouseRef-8 v2 Expression beadchips (Illumina, San Diego, CA) following protocols listed on the Gene Expression and Genomics Unit of the NIA (<http://www.grc.nia.nih.gov/branches/rrb/dna/index/protocols.htm>). The fluorescence signals were extracted using an Illumina BeadArray 500GX reader and normalized by log z-transformation to obtain z-scores and distribution tests, as previously described ([Cheadle *et al.* 2003](#_ENREF_1)). Correlation analysis, sample clustering analysis and principal component analysis including all probes was performed to identify/exclude any possible outliers. The resulting dataset was next analyzed with DIANE 6.0, a spreadsheet-based microarray analysis program. Gene set enrichment analysis used gene expression values or gene expression change values for all of the genes on the microarray. Parametric analysis of gene set enrichment (PAGE) was used for gene set analysis ([De *et al.* 2010](#_ENREF_2)). Gene sets include the MSIG database [[Link](http://www.broadinstitute.org/gsea/msigdb/collection_details.jsp#C2)], Gene Ontology Database [[Link](http://www.geneontology.org/)], GAD human disease and mouse phenotype gene sets were used to explore changes at the functional level ([Zhang *et al.* 2010](#_ENREF_4)). A subset of gene expression changes was verified by quantitative real-time PCR (Table S7).

***RT-qPCR*** Liver total RNA was isolated as described above and first strand cDNA was synthesized using the High Capacity cDNA reverse transcription kit (Applied Biosystems, Foster City, CA) according to manufacturer's instructions. Real-time quantitative PCR was performed with SYBR® Green PCR master mix on an ABI Prism 7300 sequence detection system (Applied Biosystems). Each sample was analyzed in triplicate and relative gene expression was normalized to GAPDH. The data were analyzed using the 2^−ΔΔ^*^CT^* method. Primer sequences are listed in Table S8.

***Western blotting*** Frozen liver tissues were homogenized in radioimmune precipitation buffer (Boston BioProducts, Ashland, MA). Samples were then centrifuged, and protein levels were determined by the Bradford assay (Bio-Rad Laboratories, Hercules, CA). Proteins were separated by SDS-PAGE and transferred onto nitrocellulose membranes and signals were detected by using the ECL Plus Western blotting detection system (GE Healthcare, Pascataway, NJ). The quantification was done by volume densitometry using ImageJ software and normalized to Ponceau S (Sigma-Aldrich, St. Louis, MO). Polyclonal SIRT1 and MUP antibodies were purchased from EMD-Millipore Corp. (Billerica, MA) and Santa Cruz (Dallas, TX) respectively.

***Statistical analysis*** Data are expressed as mean ± SEM. Data were analyzed using one-way ANOVA followed by Bonferroni's Post Hoc test. P-values for survival analyses were calculated using the log rank test. A difference with p-values less than 0.05 were considered statistically significant. Statistical analyses were performed using SPSS for Windows (version 17.0; SPSS, Inc., Chicago, IL) or using GraphPad Prism version 4.0 software.

**References**

Cheadle C, Cho-Chung YS, Becker KG, Vawter MP (2003). Application of z-score transformation to Affymetrix data. *Applied bioinformatics*. **2**, 209-217.

McBurney MW, Yang X, Jardine K, Hixon M, Boekelheide K, Webb JR, Lansdorp PM , Lemieux M (2003). The mammalian SIR2alpha protein has a role in embryogenesis and gametogenesis. *Molecular and cellular biology*. **23**, 38-54.

Zhang Y, De S, Garner JR, Smith K, Wang SA, Becker KG (2010). Systematic analysis, comparison, and integration of disease based human genetic association data and mouse genetic phenotypic information. *BMC Med Genomics*. **3**, 1.

**Table S1.** Numbers and survival characteristics of SIRT1^+/+^, SIRT1^+/-^ and SIRT1^-/-^ ad libitum (AL) and calorie-restricted (CR) mice.

| Genotype | Diet | N  (Censored^1^) | N  (F) | N  (M) | Median^2^  (F) | Median^2^  (M) | Median^2^  All | Oldest^2^ 20% |
| --- | --- | --- | --- | --- | --- | --- | --- | --- |
| SIRT1^+/+^ | AL | 16 (7) | 7 (4) | 9 (3) | N/A | 108 | 109 | N/A |
| SIRT1^+/-^ | AL | 15 (6) | 7 (2) | 8 (4) | 106 | 95 | 102 | N/A |
| SIRT1^-/-^ | AL | 14 (1) | 7 | 7 (1) | 66 | 62 | 64 | N/A |
| SIRT1^+/+^ | CR | 13 | 9 | 4 | 175 | 151 | 165 | 200 |
| SIRT1^+/-^ | CR | 18 | 8 | 10 | 157 | 130 | 155.5 | 179 |
| SIRT1^-/-^ | CR | 12 | 7 | 5 | 93 | 73 | 83 | 102 |

^(1)^ Censored after passing the median survival for tissue collection.

^(2)^ Units are represented in weeks.

**Table S2.** Significance for all pairwise comparisons (diet, genotype and sex) using log rank test.

| **Comparison** | **p-value Based on Log rank Test** | |
| --- | --- | --- |
|  | **Univariate** | **Stratified by Gender** |
| SIRT1^+/+^: AL vs. CR | 0.009 | 0.019 |
| SIRT1^+/-^: AL vs. CR | 0.077 | 0.085 |
| SIRT1^-/-^: AL vs. CR | 0.39 | 0.48 |
| SIRT1^+/+^ vs. SIRT1^+/-^ vs. SIRT1^-/-^  (all with AL diet) | 0.014 (test for trend) | 0.017 (test for trend) |
| SIRT1^+/+^ vs. SIRT1^+/-^ vs. SIRT1^-/-^  (all with CR diet) | <0.001 (test for trend) | <0.001 (test for trend) |
| *SIRT1^+/+^ vs. SIRT1^+/-^ (all with AL diet)* | *0.69* | *0.70* |
| *SIRT1^+/+^ vs. SIRT1^-/-^ (all with AL diet)* | *0.008* | *0.009* |
| *SIRT1^+/-^ vs. SIRT1^-/-^ (all with AL diet)* | *0.035* | *0.050* |
| *SIRT1^+/+^ vs. SIRT1^+/-^ (all with CR diet)* | *0.11* | *0.28* |
| *SIRT1^+/+^ vs. SIRT1^-/-^ (all with CR diet)* | *<0.001* | *<0.001* |
| *SIRT1^+/-^ vs. SIRT1^-/-^ (all with CR diet)* | *<0.001* | *<0.001* |

**Table S3.** Z-scores of top 100 genes altered by CR and sorted against SIRT1^-/-^ mice.

|  |  | Z-score |  |
| --- | --- | --- | --- |
| Gene | CR:AL (+/+) | CR:AL (+/-) | CR:AL (-/-) |
| Cyp2b9 | 9.69 | 17.7 | 16.16 |
| Cyp2b23 | 9.89 | 13.56 | 13.97 |
| Upp2 | 3.23 | 5.22 | 9.96 |
| OTTMUSG00000000231 | -14.95 | -12.52 | 8.67 |
| Apoc2 | -2.23 | 8.81 | 7.97 |
| Psmd8 | 2.28 | 13.71 | 7.74 |
| Slco1a4 | 9.12 | 7.12 | 6.67 |
| Acot3 | 11.05 | 6.71 | 5.99 |
| Ranbp3l | 4.19 | 5.07 | 5.47 |
| Bdh2 | 3.43 | 19.66 | 5.32 |
| Slc2a2 | 1.5 | 4.37 | 5.17 |
| Por | 6.7 | 4.67 | 4.83 |
| Hsd17b6 | 2.92 | 8.67 | 4.35 |
| Foxq1 | -1.1 | 5.04 | 3.76 |
| Abca8a | -0.67 | 4.98 | 3.47 |
| Dhdh | -1.22 | 4.78 | 3.4 |
| Cyp2c37 | 6.33 | 5.01 | 3.13 |
| Prei4 | 1.62 | 5.77 | 3.12 |
| Slc38a4 | 1.73 | 5.45 | 3.06 |
| Atp6v1d | 1.94 | 5.49 | 3.01 |
| Aacs | -3.77 | -4.64 | 2.95 |
| Lgals4 | 2.91 | 6.5 | 2.85 |
| Accn5 | 0.36 | 6.49 | 2.53 |
| Zfp97 | 1.78 | 4.51 | 2.4 |
| Cyp39a1 | 6.52 | 4.71 | 2.14 |
| Acot4 | 4.16 | 8.16 | 2.13 |
| Sort1 | 1.15 | 5.31 | 2.1 |
| Serpina12 | -10.26 | 4.78 | 2.08 |
| LOC100044862 | -2.27 | 6.62 | 2.07 |
| EG241041 | -6.01 | 7.02 | 2.06 |
| Spred1 | 2.74 | 4.67 | 1.97 |
| Esr1 | 1.84 | 4.35 | 1.92 |
| Cyp4a14 | 17.23 | 8.17 | 1.67 |
| Mrps12 | 1.43 | 4.7 | 1.5 |
| Gm106 | -4.94 | -6.36 | 1.49 |
| 9030612M13Rik | -1.2 | 5 | 1.42 |
| Hsd3b5 | -18.25 | -7.3 | 1.28 |
| Ddx3y | -7.39 | -5.38 | 1.23 |
| Cyp2d9 | -10.06 | -6.45 | 1.23 |
| Gstp1 | -4.93 | -4.9 | 1.22 |
| Cyp3a11 | 7.46 | 7.19 | 1.14 |
| Eif4ebp3 | 4.04 | 6.52 | 1.08 |
| Cpsf4l | 1.5 | 6.09 | 0.94 |
| Fxyd6 | 0.5 | 4.85 | 0.94 |
| Slc39a4 | -0.66 | 8.15 | 0.93 |
| Wsb1 | 3.7 | 4.41 | 0.76 |
| Cish | -0.74 | 7.68 | 0.73 |
| Krt23 | 5.12 | 9.92 | 0.58 |
| EG624219 | -5.33 | -5.67 | 0.47 |
| Cyp4a12a | -25.84 | -10.32 | 0.36 |
| Cyp4a12b | -10.48 | -7.11 | 0.06 |
| Gdf10 | 1.68 | 4.53 | 0.05 |
| Ccbl2 | 4.83 | 6.6 | 0.04 |
| Cyp2u1 | -5.46 | -4.86 | 0.02 |
| Slc38a5 | 6.3 | 4.84 | -0.02 |
| Ela1 | -6.05 | -5.14 | -0.25 |
| Ephx1 | -2.19 | -4.52 | -0.27 |
| Elovl3 | -10.77 | -7.76 | -0.55 |
| Irs2 | 7.27 | 4.85 | -0.6 |
| Rusc2 | 0.79 | 6.42 | -0.6 |
| Cyp7b1 | -7.94 | -5.59 | -0.67 |
| Il6st | 1.84 | 4.98 | -1.34 |
| C6 | -9.7 | -5.38 | -1.35 |
| Gdf15 | -3.03 | -4.44 | -1.61 |
| Phlda1 | -3.02 | 5.13 | -1.64 |
| Alas2 | -7.26 | -5.87 | -1.65 |
| Fcgr4 | -1.19 | -4.51 | -1.75 |
| Mup4 | -12.55 | -7.34 | -2.16 |
| Igsf11 | -0.45 | -4.92 | -2.24 |
| Mvd | -1 | -7.31 | -2.26 |
| Nsdhl | -0.37 | -5.42 | -2.33 |
| Aqp8 | -8.02 | -7.79 | -2.47 |
| Fdps | -0.85 | -4.9 | -2.58 |
| Tubb2c | -2.38 | -7.36 | -2.63 |
| Nnmt | 5.53 | 7.1 | -2.94 |
| Dct | -6.56 | 7.68 | -2.99 |
| Chac1 | -3.78 | 6.92 | -3 |
| H2-Bl | -9.51 | 4.97 | -3 |
| Tuba6 | -3.28 | -6.66 | -3.08 |
| Cyp51 | 0.32 | -5.76 | -3.44 |
| Sae1 | -3.14 | -4.87 | -3.48 |
| Tuba1b | -2.97 | -7.72 | -3.66 |
| Gck | -10.06 | -5.06 | -3.81 |
| Pdzrn3 | 2.33 | 4.39 | -3.91 |
| OTTMUSG00000007485 | -17.62 | -10.81 | -4.02 |
| LOC100040592 | -2.11 | -6.49 | -4.19 |
| Tfb1m | -4.35 | 4.56 | -4.19 |
| Sqle | -0.97 | -7.87 | -4.45 |
| G6pc | 2.99 | 4.66 | -4.59 |
| Mup1 | -12.09 | -6.15 | -4.81 |
| Slc30a10 | -2.46 | -6.59 | -4.88 |
| Cyp2a12 | -2.31 | -6.92 | -5.12 |
| Tubb2b | -2.42 | -10.3 | -5.16 |
| Gale | 1.23 | -5.41 | -5.18 |
| LOC620807 | -13.58 | -5.73 | -5.18 |
| Fgf21 | -2.96 | -6.47 | -5.34 |
| Tsc22d1 | -5.85 | -6.07 | -5.38 |
| Paqr9 | -3.51 | -4.41 | -5.99 |
| Mup2 | -14.74 | -12.22 | -6.04 |
| Spon2 | -7.14 | -5.62 | -7.65 |
|  |  |  |  |
|  |  |  |  |
|  |  |  |  |

**Table S4.** List of genes affected by CR in a SIRT1-independent manner.

|  |  | Z-score |  |
| --- | --- | --- | --- |
| Gene | CR:AL | CR:AL | CR:AL |
|  | (+/+) | (+/-) | (-/-) |
| Gm129 | 1.12 | -1.66 | 9.93 |
| Akr1c19 | 2.68 | -1.69 | 8.7 |
| Olfml1 | -1.15 | -1.24 | 5.31 |
| Cyp2j9 | -0.16 | 0.09 | 5.04 |
| 4833442J19Rik | 1.41 | 0.23 | 4.85 |
| Pitpnc1 | 1.19 | 1.25 | 4.44 |
| Ngef | 1.92 | 2.54 | 3.96 |
| Ociad2 | -1.96 | 0.6 | 3.96 |
| Fmo5 | 1.29 | 1.99 | 3.89 |
| Acbd5 | 1.19 | 0.49 | 3.72 |
| Slc47a1 | 2.04 | -2.17 | 3.63 |
| 1300013J15Rik | 2.11 | -2.37 | 3.48 |
| Slc7a4 | 1.32 | 1.34 | 3.42 |
| Lrrc28 | -1.51 | -1.77 | 3.39 |
| Slc6a6 | -0.6 | 0.07 | 3.39 |
| Cobll1 | 1.47 | 0.23 | 3.26 |
| Gcnt2 | 0 | 0.24 | 3.2 |
| Ces5 | 0.82 | 1.41 | 3.16 |
| 2410129H14Rik | 0.78 | 0.59 | 3.01 |
| Adamts7 | 1.2 | 2.11 | 2.88 |
| A330080J22Rik | 1.37 | 1.64 | 2.87 |
| Adarb1 | 1.06 | 1.26 | 2.87 |
| Vegfb | 0.53 | 0.47 | 2.74 |
| LOC100047674 | -0.67 | 0.94 | 2.73 |
| Pgrmc1 | 0.2 | 1.64 | 2.7 |
| Mef2c | 1.5 | 1.82 | 2.67 |
| Uvrag | -0.17 | -0.87 | 2.66 |
| Ppp2r5e | -0.16 | 0.42 | 2.61 |
| Aph1a | 0.95 | 0.51 | 2.49 |
| Pfkm | 0.91 | 0.45 | 2.49 |
| Slc44a1 | 0.71 | 0.58 | 2.49 |
| Ankrd12 | 1.46 | 1.05 | 2.46 |
| Cryz | -0.09 | 1.89 | 2.43 |
| Cd99l2 | 0.79 | 0.66 | 2.41 |
| L2hgdh | 0.05 | 0.28 | 2.38 |
| Acaa2 | 0.97 | 1.1 | 2.36 |
| Ctnnbip1 | 0.36 | 0.71 | 2.36 |
| 1500001M20Rik | 1.09 | 0.75 | 2.33 |
| Acot7 | 1.47 | 0.59 | 2.32 |
| Cyb5r3 | -0.87 | -0.61 | 2.32 |
| Elf2 | 1.43 | 1.37 | 2.28 |
| Slc9a3r2 | 0.02 | 0.35 | 2.28 |
| 5230400G24Rik | 1.36 | 0.51 | 2.23 |
| Foxn3 | -0.74 | 1.26 | 2.23 |
| Ak2 | -0.62 | -0.88 | 2.22 |
| 6430510M02Rik | 0.53 | 0.69 | 2.19 |
| Zbtb38 | 1.48 | 1.4 | 2.18 |
| Pcgf3 | 0.12 | 1.02 | 2.17 |
| Grasp | 0.78 | 0.62 | 2.16 |
| Ddt | 1.48 | 2.55 | 2.14 |
| Ccdc3 | -0.12 | 1.48 | 2.13 |
| Cdc23 | 1.32 | 0.86 | 2.11 |
| 6330578E17Rik | 1.2 | 1.21 | 2.09 |
| Arpc1b | 1.26 | 1.35 | 2.08 |
| Add3 | 1.13 | 1.26 | 2.05 |
| Vegfa | 0.69 | 1.35 | 2.05 |
| Renbp | 1.36 | 0.62 | 2.03 |
| Tex261 | 1.06 | 1.31 | 2.02 |
| Cul3 | 0.61 | 0.99 | 1.98 |
| Pcgf5 | 1.46 | 1.34 | 1.98 |
| Stard7 | 0.42 | 1.02 | 1.98 |
| Trim8 | 0.58 | -0.67 | 1.96 |
| Csnk2a2 | 1.23 | 0.85 | 1.95 |
| Klf15 | 1.8 | -0.66 | 1.94 |
| Myo10 | -0.73 | -0.35 | 1.93 |
| Stk35 | 1.28 | 2 | 1.91 |
| Zfp771 | 0.7 | -0.63 | 1.91 |
| Mdh1 | 0.65 | 1.27 | 1.9 |
| Rsn | 0.62 | -0.63 | 1.9 |
| Col4a3bp | 0.44 | 0.42 | 1.89 |
| Traf4 | 0.74 | -0.59 | 1.88 |
| Gtf2ird2 | 1.27 | 0.25 | 1.86 |
| Ifrd2 | 1.12 | 0.16 | 1.86 |
| Cpeb3 | 1.2 | -0.32 | 1.83 |
| Tmem134 | 1.25 | 1.43 | 1.83 |
| Rsrc1 | 0.91 | 0.94 | 1.82 |
| Atxn10 | 1.03 | 1.57 | 1.79 |
| C80913 | 1 | 0.94 | 1.79 |
| LOC100048020 | 1.07 | 1.31 | 1.77 |
| 5730596K20Rik | 1.22 | 0.19 | 1.76 |
| Centg3 | -0.01 | -0.18 | 1.76 |
| Tmem204 | 1.2 | 1.31 | 1.76 |
| Ppic | 0.42 | 1.2 | 1.75 |
| Esam1 | 0.59 | 0.75 | 1.74 |
| Arih2 | 0.44 | 0.01 | 1.73 |
| Cdk8 | 1.28 | 0.47 | 1.73 |
| Rragc | 0.25 | 0.12 | 1.73 |
| 4631426J05Rik | 0.75 | 0.77 | 1.72 |
| Mbtd1 | 0.58 | 1.41 | 1.7 |
| Myl4 | 0.98 | -0.04 | 1.7 |
| LOC100047837 | 1.28 | 0.43 | 1.67 |
| Crebl2 | 1.29 | 0.55 | 1.66 |
| Pik3cb | 0.98 | 1.62 | 1.66 |
| Reep3 | 0.27 | 0.9 | 1.66 |
| Vamp3 | 0.99 | 0.2 | 1.66 |
| Bnip3l | 1.17 | 0.95 | 1.65 |
| Msi2 | 0.3 | -0.06 | 1.65 |
| Styx | 1.5 | 0.52 | 1.64 |
| 1810020D17Rik | -1.38 | 0.94 | 1.62 |
| Rbm47 | 0.33 | 0.63 | 1.61 |
| Ubr5 | 0.93 | 0.77 | 1.6 |
| Cnot7 | 1.37 | 0.14 | 1.59 |
| Foxp1 | 0.33 | 1.37 | 1.58 |
| Ldb2 | 0.49 | 1.48 | 1.56 |
| Zxdc | 0.57 | 1 | 1.56 |
| Unc5b | 0.09 | 1.79 | 1.54 |
| Trappc1 | -0.5 | -1.02 | -1.5 |
| Unc119b | -1.24 | -0.72 | -1.5 |
| 9430023L20Rik | 0.65 | -0.09 | -1.53 |
| Gtf3c5 | -0.36 | -0.07 | -1.56 |
| Med10 | -0.37 | -1 | -1.56 |
| Slc29a3 | -1.07 | -0.27 | -1.56 |
| Wdr13 | -1.47 | -1.07 | -1.58 |
| Flad1 | -1.21 | -1.13 | -1.59 |
| Uba1 | -0.95 | -1.48 | -1.61 |
| Trp53bp1 | -0.58 | -0.52 | -1.64 |
| Rab7l1 | -1.41 | -0.89 | -1.66 |
| Snrpa | -0.39 | -1.23 | -1.75 |
| Hist1h2bk | -1.6 | -1.09 | -1.83 |
| Pomgnt1 | -0.6 | -0.36 | -1.85 |
| Ap3s2 | -1.02 | -0.63 | -1.87 |
| Sdf2 | -0.98 | -1.19 | -1.87 |
| Trp53 | -0.5 | -0.13 | -1.9 |
| Ccdc123 | -0.22 | 0.28 | -1.92 |
| Hist1h4h | 0.1 | 0.96 | -1.93 |
| Orai3 | -0.14 | 0.15 | -1.93 |
| Sigirr | 0.6 | -1.34 | -1.94 |
| Lcat | -0.22 | -0.79 | -1.95 |
| Mnat1 | -1.14 | -0.33 | -1.96 |
| Tspan33 | -1.47 | -1.41 | -1.96 |
| Dcakd | -0.49 | -1.19 | -2.04 |
| Gtf2h3 | -0.12 | 0.65 | -2.04 |
| Ap3d1 | -1.04 | -0.9 | -2.05 |
| Apof | -1.38 | -0.64 | -2.05 |
| Mrrf | -0.3 | -0.25 | -2.06 |
| Dalrd3 | -1.17 | -0.52 | -2.07 |
| Diablo | -0.71 | -0.46 | -2.09 |
| Hist1h2bn | -1.16 | -0.21 | -2.13 |
| Itgb3bp | 0.24 | 1.37 | -2.13 |
| Lgtn | -1.34 | -1.42 | -2.13 |
| Ppp1r11 | -0.46 | -0.97 | -2.15 |
| Twf2 | 0.26 | -1.36 | -2.15 |
| Mpv17 | 0.19 | 0.45 | -2.17 |
| Myd88 | -0.22 | -0.93 | -2.17 |
| BC006779 | -0.58 | -0.28 | -2.21 |
| Sra1 | -0.73 | -1.2 | -2.34 |
| B3gat3 | -1.04 | -1.61 | -2.37 |
| Cops6 | -0.53 | -0.96 | -2.38 |
| Hist1h4i | -0.92 | 0.81 | -2.41 |
| Hist1h2bm | -1.34 | -1.15 | -2.42 |
| Hist1h4j | -0.21 | 0.9 | -2.63 |
| Bckdk | -1.18 | -1.06 | -2.75 |
| Mustn1 | 1.16 | -0.26 | -2.79 |
| Ears2 | -0.19 | 0.27 | -2.82 |
| F11 | -1.78 | -0.51 | -2.82 |
| 1810009A15Rik | -1.29 | -0.9 | -2.94 |
| Pdia5 | 1.15 | 0.77 | -2.98 |
| Uros | -0.97 | -0.07 | -3.08 |
| Pla2g12b | -0.32 | -1.14 | -3.12 |
| Wdr46 | -3.59 | 1.35 | -3.12 |
| AU022252 | -1.65 | -1.45 | -3.15 |
| BC017612 | -0.34 | -1.23 | -3.16 |
| Adck5 | -1.28 | 0.88 | -3.17 |
| Dpp9 | -0.16 | -1.19 | -3.2 |
| A030007L17Rik | -1.04 | -0.7 | -3.36 |
| Tbrg1 | -1.06 | -1.45 | -3.38 |
| Tnfaip2 | -0.72 | -0.2 | -3.48 |
| Ang | 0.13 | -0.83 | -3.71 |
| Pes1 | -0.67 | 0.98 | -3.78 |
| Fpgs | -1.37 | -1.1 | -3.92 |
| Avpr1a | 0.72 | -0.59 | -4.57 |
| Cldn1 | -0.89 | 1.52 | -6.67 |
| Lrfn3 | 0.47 | -0.98 | -7.19 |
| Cyp17a1 | 2.74 | 0.64 | -9.39 |
|  |  |  |  |
|  |  |  |  |
|  |  |  |  |
|  |  |  |  |
|  |  |  |  |
|  |  |  |  |
|  |  |  |  |

**Table S5.** RT-qPCR validation of genes affected by CR in a SIRT1-independent manner.

|  | mRNA expression | | |
| --- | --- | --- | --- |
| Genes | CR:AL (+/+) | CR:AL (+/-) | CR:AL (-/-) |
| ANG | 0.99 ± 0.19^*^ | 0.88 ± 0.27 | 0.35 ± 0.07 |
| ADCK5 | 0.53 ± 0.09 | 1.05 ± 0.06^*^ | 0.40 ± 0.02 |
| CYP17A1 | 1.15 ± 0.43 | 0.62 ± 0.21 | 0.15 ± 0.05 |
| AKR1c19 | 1.13 ± 0.34^*^ | 1.12 ± 0.32 | 3.45 ± 0.12 |
| ANKRD12 | 0.71 ± 0.12 | 0.98 ± 0.06 | 1.11 ± 0.10 |
| CYP2J9 | 0.38 ± 0.09 | 0.86 ± 0.05^*^ | 0.63 ± 0.07 |
| FMO5 | 0.66 ± 0.16^*^ | 1.11 ± 0.16 | 2.24 ± 0.43 |
| MEF2c | 0.54 ± 0.08^*^ | 0.89 ± 0.07^*^ | 1.16 ± 0.03 |
| TNFAIP2 | 0.52 ± 0.02^*^ | 0.99 ± 0.15^*^ | 0.29 ± 0.04 |

mRNA levels were measured by RT-qPCR in liver homogenates from ad libitum (AL) and 40% CR-fed SIRT1^+/+^, SIRT1^+/-^ and SIRT1^-/-^ mice. n=3 for all groups (*p<0.05 compared to SIRT^-/-^). Error bars indicate SEM.

**Table S6.** Pathologies and abnormalities in old mice (> 24 months)

| CR | Gender | Survival(weeks) | Pathology (observations after 2 years) |
| --- | --- | --- | --- |
| SIRT1^+/+^ | F | 175 | Growth in the left eye, and in the cheek |
|  | F | 87 |  |
|  | M | 123 |  |
|  | F | 180 |  |
|  | F | 128 |  |
|  | F | 170 | Lost fur |
|  | F | 213 |  |
|  | M | 147 | Left eye problem |
|  | M | 165 | Both eyes have infection, left eye is severely damaged |
|  | F | 176 |  |
|  | F | 206 |  |
|  | F | 156 |  |
|  | M | 155 |  |
|  |  |  |  |
| SIRT1^+/-^ | F | 168 | Back legs problem. Mouse can't move well |
|  | F | 162 |  |
|  | F | 109 |  |
|  | F | 158 | Infection in both eyes |
|  | M | 103 |  |
|  | M | 107 |  |
|  | M | 180 | Left eye is enlarged |
|  | M | 78 |  |
|  | F | 156 |  |
|  | F | 182 | Abnormal growth in the middle of both eyes |
|  | F | 143 |  |
|  | F | 155 | Abnormal growth in right ear (possibly tumor) |
|  | M | 156 |  |
|  | M | 175 | Redness in both eyes (partial bleeding) |
|  | M | 83 |  |
|  | M | 160 | Eye abnormality |
|  | M | 153 | Eye abnormality |
|  | M | 82 |  |

**Table S7.** RT-qPCR validation of microarray data

|  | Z-score (Array) | | | Fold change (RT-qPCR) | | |
| --- | --- | --- | --- | --- | --- | --- |
| Gene Symbol | CR:AL (+/+) | CR:AL (+/-) | CR:AL  (-/-) | CR:AL (+/+) | CR:AL (+/-) | CR:AL  (-/-) |
| Eif4ebp3 | 4.04 | 6.52 | 1.08 | 1.41 | 2.70 | 1.05 |
| Gstt3 | 3.57 | 3.24 | 2.49 | 2.57 | 2.51 | 1.41 |
| Slco1a4 | 9.12 | 7.12 | 6.67 | 9.35 | 5.16 | 1.05 |
| Cyp39a1 | 4.56 | 2.58 | 1.04 | 8.69 | 5.09 | -1.09 |
| Utx | 3.79 | 3.04 | 1.56 | 1.22 | 1.36 | -1.80 |
| Mx2 | -3.91 | -2.65 | -1.03 | -4.87 | -1.07 | -2.23 |
| Gm106 | -4.94 | -6.36 | 1.49 | -2.42 | 1.18 | -3.10 |
| Rdh20 | -1.81 | -2.24 | 0.30 | -1.73 | -1.13 | -1.32 |
| Aqp8 | -8.02 | -7.79 | -2.47 | -8.82 | -2.87 | -2.00 |
| Derl2 | -3.01 | -1.73 | -1.49 | -2.04 | -1.35 | -1.46 |

Liver RNA samples from AL and 40% CR-fed SIRT1^+/+^, SIRT1^+/-^ and SIRT1^-/-^ mice were amplified using primers specific for the indicated targets. Targets were selected based on the common genes between SIRT1^-/-^ and SIRT1^+/-^. The numbers in red did not match the Z-scores of the microarray data.

**Table S8.** Primer sequences used for quantitative PCR analysis

| Gene | Sense | Antisense |
| --- | --- | --- |
| SIRT1 | CAGCAACACCTCATGATTGG | TCCGACAGGAAACAGAAACC |
| Eif4ebp3 | CCAGCAGCCTTGGTTAAGAG | AGCTGCTGGAACAGTCCAAT |
| Gstt3 | TCCTGAAGGCCAAGGATATG | CACAGGTGATGCAGAGAGGA |
| Slco1a4 | GCAGTCATTAAGCCCAGAGC | GCCAACAGAAATGCCTTGAT |
| Cyp39a1 | CAGTGTCCTGGAAGGTGGTT | GGCACGTAGCTTCACTCCTC |
| Utx | ATGGAAACGTGCCTTACCTG | GGACCTGCCAAATGTGAACT |
| Mx2 | CCCAGAGGCAGTGGTATTGT | ACATTTGGGGAGCTGACATC |
| Gm106 | GGGACTGCTGCTTCGACTAC | CAGTCCTGGCTCTGTGATGA |
| Rdh20 | CCTCCACAGGTACCAAGCAT | TTCCTGGGGTTTGTTTCTTG |
| Aqp8 | GCAGGACCTGAGCTAACAGG | GCATTCTCCACCTTCTCAGC |
| Derl2 | AGAAGGCTCTTTCCGAGGTC | GGGGCCTGGAAGTTTAGAAG |
| Mup1 | AACGAAAAGGATGGGGAAAC | GGCAGCGATCTGTAGTGTGA |
| Gck | CTTTCCAGGCCACAAACATT | TGAGTGTTGAAGCTGCCATC |
| Irs2  Cyp17a1  Cyp2j9  Tnaip2  Ankrd12  Akr1c19  Fmo5  Mef2c | GTAGTTCAGGTCGCCTCTGC  TTTTGGCCCAAGTCAAAGAC  ATCTGCAGCACTCTGGACCT  AAAAAGGACCAGCCCAGATT  AGCTGGAGGGTCTCACTGAA  AGCTCTAGAGGCTGGGTTCC  ACAGGGCTCTGAGTCAGCAT  CGGTGTCGTCAGTTGTATGC | CAGCTATTGGGACCACCACT  CCCTTCTTCACGAGCACTTC  CTCATGGATGACAGCATTGG  TACAGAGCCTCCACCTTGCT  CAGGTCTCTCTGGGAACAGC  CAGCTCTGGCCTATGGAAAG  TGTGGCAAAGATGACCACAT  TGCAGTAGATATGCGGCTTG |
| Adck5  Ang | CCCTGCCATATTCTTCAGGA  AACCTCACCCTGCAAAGATG | ACGTTTGTGCACCACCAGTA  AGTGGACAGGCAAACCATTC |
| GAPDH | CACCAACTGCTTAGCCCC | TCTTCTGGGTGGCAGTGATG |

**Supplementary Figure legend**

**Figure S1.** Parametric analysis of gene-set enrichment (PAGE) was performed on microarray data from SIRT1^+/+^, SIRT1^+/-^ and SIRT1^-/-^ mice fed ad libitum (AL) or subjected to 40% CR. Columns show genes (A) and pathways (B) significantly up- (red) or down-regulated (blue) by CR when sorted against the profile obtained in SIRT1^-/-^ mice.

**Figure S2.** Genes affected by CR in a SIRT1 gene-dose dependent manner. (A) IRS2 GCK, and MUP1 mRNA levels were measured by RT-qPCR in liver homogenates from ad libitum (AL) and 40% CR-fed SIRT1^+/+^, SIRT1^+/-^ and SIRT1^-/-^ mice. n=3 for all groups (*p<0.05 compared to SIRT1^+/+^). Error bars indicate SEM. (B) MUP1 protein levels were determined by Western blotting. n=2-3 for all groups.

**Fig. S1.**

**A B**

**
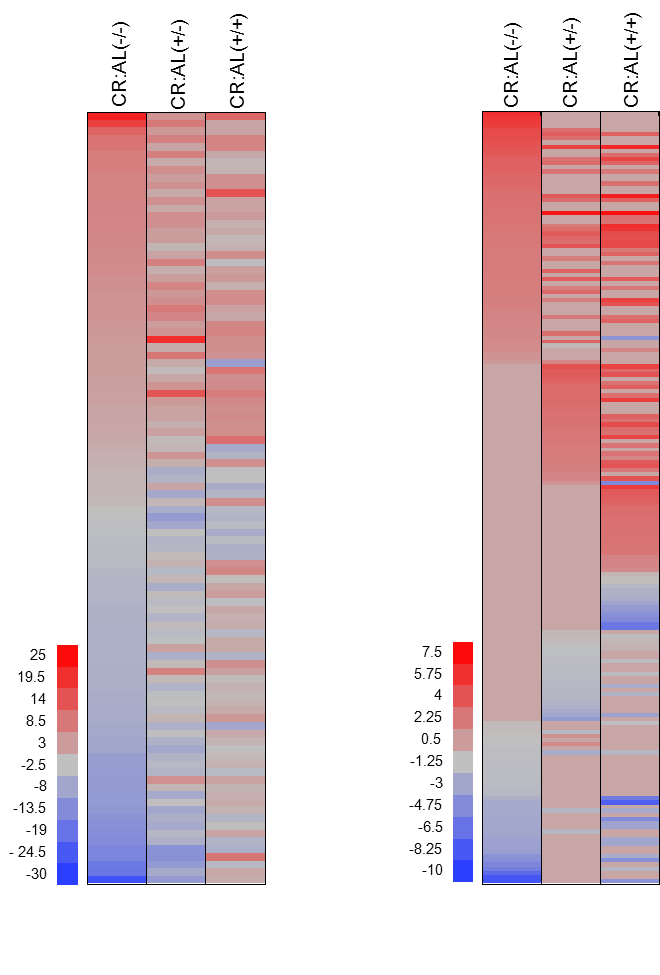
**

**Fig. S2.**

**A.**

*


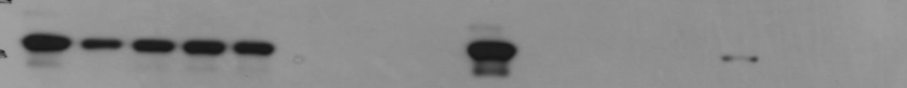
**B.**

+/+

+/-

-/-

+/+

+/-

-/-

MUP1

AL CR

Poinceau S
